# Prognostic Significance of Plasma Neutrophil Extracellular Trap Levels in Patients with Non-Small Cell Lung Cancer Treated with Immune Checkpoint Inhibitors

**DOI:** 10.3390/biomedicines12081831

**Published:** 2024-08-12

**Authors:** Shun Horaguchi, Yoshiro Nakahara, Yuka Igarashi, Taku Kouro, Feifei Wei, Kenta Murotani, Seiichi Udagawa, Naoko Higashijima, Norikazu Matsuo, Shuji Murakami, Terufumi Kato, Tetsuro Kondo, Huihui Xiang, Rika Kasajima, Hidetomo Himuro, Kayoko Tsuji, Yasunobu Mano, Mitsuru Komahashi, Yohei Miyagi, Haruhiro Saito, Koichi Azuma, Shuichiro Uehara, Tetsuro Sasada

**Affiliations:** 1 Cancer Vaccine and Immunotherapy Center, Kanagawa Cancer Center Research Institute, Yokohama 241-8515, Japan; shun.horaguchi701@gmail.com (S.H.); kouro.3v70h@kanagawa-pho.jp (T.K.); feifei.wei@gancen.asahi.yokohama.jp (F.W.); h-himuro@kcch.jp (H.H.); kayokotsuji@gancen.asahi.yokohama.jp (K.T.); 2022ymano07@gmail.com (Y.M.); mi.komahashi@gancen.asahi.yokohama.jp (M.K.); 2 Division of Cancer Immunotherapy, Kanagawa Cancer Center Research Institute, Yokohama 241-8515, Japan; adipomin@gmail.com (Y.I.); acorn5083.nao@gmail.com (N.H.); 3 Department of Pediatric Surgery, Nihon University School of Medicine, Tokyo 173-8610, Japan; 4 Department of Thoracic Oncology, Kanagawa Cancer Center, Yokohama 241-8515, Japan; md100062@kitasato-u.ac.jp (Y.N.); murakamis@kcch.jp (S.M.); katote@kcch.jp (T.K.); kondot@kcch.jp (T.K.); saito-h@kcch.jp (H.S.); 5 Department of Respiratory Medicine, Kitasato University School of Medicine, Sagamihara 252-0375, Japan; 6 Biostatistics Center, Kurume University School of Medicine, Kurume 830-0011, Japan; kmurotani@med.kurume-u.ac.jp; 7 Mathematics Section, Division of Natural Sciences, Nihon University School of Medicine, Tokyo 173-0032, Japan; udagawa.seiichi@nihon-u.ac.jp; 8 Division of Respirology, Neurology, and Rheumatology, Department of Internal Medicine, Kurume University School of Medicine, Kurume 830-0011, Japan; matsuo_norikazu@med.kurume-u.ac.jp (N.M.); azuma@med.kurume-u.ac.jp (K.A.); 9 Molecular Pathology and Genetics Division, Kanagawa Cancer Center Research Institute, Yokohama 241-8515, Japan; xiang@gancen.asahi.yokohama.jp (H.X.); rkasajima@gancen.asahi.yokohama.jp (R.K.); miyagi.0e82r@kanagawa-pho.jp (Y.M.)

**Keywords:** neutrophil extracellular trap (NET), immune checkpoint inhibitor, anti-PD-1 antibody, anti-PD-L1 antibody, non-small cell lung cancer (NSCLC)

## Abstract

Neutrophil extracellular traps (NETs) released from neutrophils are related to cancer progression. However, the relationship between the therapeutic effects of immune checkpoint inhibitors (ICIs) such as anti-PD-1 and anti-PD-L1 antibodies and plasma NET concentration in patients with non-small cell lung cancer (NSCLC) is poorly understood. In this study, concentrations of citrullinated histone H3 (CitH3), a surrogate marker of NETs, in plasma before/after treatment were examined in patients with advanced or recurrent NSCLC undergoing ICI treatment (n = 185). The clinical significances of NET levels before/after treatment and posttreatment changes were statistically evaluated. As a result, multivariate Cox analysis showed that high NET levels before treatment were statistically significant predictors of unfavorable overall survival (OS; *p* < 0.001, HR 1.702, 95% CI 1.356–2.137) and progression-free survival (PFS; *p* < 0.001, HR 1.566, 95% CI 1.323–1.855). The Kaplan-Meier curves showed significant separation between the high- and low-NET groups in OS (*p* = 0.002) and PFS (*p* < 0.001). Additionally, high NET levels after treatment were also significantly associated with worse OS (*p* < 0.001) and PFS (*p* < 0.001) by multivariate Cox analysis. Notably, the pretreatment NET levels were significantly correlated with the plasma levels of NET-related inflammatory cytokines, such as IL-6 and IL-8, and with NET-related gene expression and immune-suppressive profile in peripheral blood mononuclear cells. Our findings suggest that NETs released from activated neutrophils might reduce the clinical efficacy of ICIs in patients with NSCLC.

## 1. Introduction

Lung cancer, predominantly non-small cell lung cancer (NSCLC), is a leading cause of cancer-related death worldwide [1]. For the treatment of patients with NSCLC, various modalities, such as surgery, chemotherapy, radiotherapy, and molecular-targeted drugs, have been employed, depending on the disease stage [2,3]. In addition, immune checkpoint inhibitors (ICIs), especially anti-PD-1 or anti-PD-L1 antibodies (Abs), have recently been established as a novel treatment strategy and reported to improve the prognosis of patients with advanced and recurrent NSCLC [4,5,6,7]. Nevertheless, the response rates to ICIs have been limited, and thus, the factors associated with the prognosis of ICI-treated patients remain to be identified [8].

Tumor-associated inflammation is implicated in tumor invasion, metastasis, and resistance to drug therapy. For example, the neutrophil-to-lymphocyte ratio (NLR), which reflects the balance between proinflammatory and anti-inflammatory responses, has recently been reported as a prognostic factor in patients with various cancers, including NSCLC [9,10]. The activation of neutrophils by inflammation leads to the release into the circulating blood of web-like structures composed of DNA–histone complexes and granule proteins, referred to as neutrophil extracellular traps (NETs) [11,12]. NETs were originally identified as a host defense system against infection, owing to their ability to rapidly trap and kill foreign pathogens [11,12]. In addition, increasing evidence has demonstrated that NETs are linked to cancer progression, metastasis, and cancer-associated thrombosis [11,12,13,14,15,16].

Notably, several studies have indicated that NETs may reduce the effectiveness of ICIs by negatively influencing anti-tumor immune cells in murine tumor models [17,18,19,20]. However, clinical investigations into the role of NETs in cancer patients undergoing immunotherapy remain limited. Han et al. demonstrated that a NET-related gene signature can predict an immunosuppressive microenvironment and the efficacy of immunotherapy in multiple tumor types [21]. Their study, however, focused on gene expression within tumors rather than in the peripheral blood. Wang et al. proposed that a risk score incorporating serum NETs, IL-8, and CRP could serve as a valuable predictive biomarker for ICI therapy in cancer patients [22]. Nevertheless, their study encompassed patients with diverse cancer types, including NSCLC, nasopharyngeal carcinoma, gastrointestinal tumors, and melanoma, each with different clinical characteristics. Additionally, very recently, serum NETs were reported to predict the efficacy of ICI treatment in NSCLC patients, but the study was limited by a small sample size (n = 31) [23]. In the current study, we investigated the clinical role of plasma NETs in a larger cohort of ICI-treated NSCLC patients (n = 185), which could strengthen the validity of the results due to the larger sample size. We demonstrated that the plasma levels of NETs before and after ICI treatment are associated with the survival of ICI-treated patients with cancer. 

## 2. Materials and Methods

### 2.1. Patients

This study included 185 patients with stage III, IV, or recurrent NSCLC who were treated with an anti-PD-1 Ab (nivolumab or pembrolizumab) or an anti-PD-L1 Ab (atezolizumab) between February 2017 and February 2021 at the Kanagawa Cancer Center (Yokohama, Japan) or Kurume University Hospital (Kurume, Japan). This study was conducted in accordance with the provisions of the Declaration of Helsinki and was approved by the Institutional Review Boards of the Kanagawa Cancer Center (approval numbers: 28-85 and 2019-131) and Kurume University (approval number: 19240). Written informed consent was obtained from all participants before their inclusion after the nature and possible consequences of this study were explained to them.

After enrollment, the clinical characteristics of patients, including sex, age, Eastern Cooperative Oncology Group (ECOG) performance status (PS), smoking history, mutations in driver genes (EGFR, ALK, or ROS1), histology, clinical stage, PD-L1 expression in tumor tissues, type of ICIs, concurrent chemotherapy, and treatment line were collected and recorded. PD-L1 expression in tumor tissues was assessed only in patients whose tissue samples were available (n = 164). Tumor mutation burden was not assessed in most patients because tissue samples were unavailable for next-generation sequencing. Complete blood counts, including white blood cell (WBC) and platelet (Plt) counts, and serum biochemistry tests, including albumin (ALB), lactate dehydrogenase (LDH), and C-reactive protein (CRP), were performed before the initiation of ICI treatment.

After assessment at baseline, eligible patients received intravenous nivolumab (3 mg/kg of body weight or 240 mg every 2 weeks), pembrolizumab (200 mg every 3 weeks), or atezolizumab (1200 mg every 3 weeks), with or without concurrent chemotherapy. The treatment continued until intolerable toxicity or disease progression was evaluated according to the Response Evaluation Criteria in Solid Tumors (RECIST) version 1.1. using chest and abdominal computed tomography (CT) and cranial CT or magnetic resonance imaging (MRI).

### 2.2. Analysis of Circulating NETs in Plasma

Peripheral blood was collected from patients into tubes containing heparin as an anticoagulant before and 6 weeks after ICI treatment initiation. Plasma was separated by centrifugation of whole-blood samples and stored frozen until analysis. The concentrations of citrullinated histone H3 (CitH3), which is a surrogate marker of NETs, before and after the initiation of ICI treatment, were measured using an enzyme-linked immunosorbent assay (ELISA, clone 11D3) kit from Cayman Chemical (Ann Arbor, MI, USA). Frozen plasma samples were thawed, diluted twofold, and assayed in duplicate in accordance with the manufacturer’s instructions. The mean of duplicate samples was used for statistical analysis.

### 2.3. Measurement of Soluble Immune Mediators in Plasma

The levels of other soluble immune mediators in plasma were measured in 98 patients whose plasma samples were available for this analysis. A bead-based multiplex assay (Bio-Plex 200 system; Bio-Rad Laboratories, Hercules, CA, USA) was used to evaluate the levels of soluble immune mediators, including cytokines, chemokines, and growth factors, in 50-μL aliquots of fourfold diluted plasma samples before the initiation of ICI therapy, except for transforming growth factor (TGF)-β1, TGF-β2, and TGF-β3, for which we used 16-fold diluted plasma after oxidation. The following 97 soluble immune mediators were measured: interleukin (IL)-1β, IL-2, IL-4, IL-5, IL-6, IL-8, IL-9, IL-10, IL-11, IL-12 (p40), IL-12 (p70), IL-13, IL-16, IL-17A, IL-17F, IL-17A/F, IL-19, IL-20, IL-21, IL-22, IL-23, IL-25, IL-26, IL-27, IL-28A, IL-29, IL-31, IL-32, IL-33, IL-34, IL-35, interferon (IFN) α2, IFNβ, IFNγ, tumor necrosis factor (TNF) α, granulocyte–macrophage colony-stimulating factor (GM-CSF), C–C motif chemokine ligand (CCL) 1, CCL2, CCL3, CCL7, CCL8, CCL11, CCL13, CCL15, CCL17, CCL19, CCL20, CCL21, CCL22, CCL23, CCL24, CCL25, CCL26, CCL27, C–X–C motif chemokine ligand (CXCL) 1, CXCL2, CXCL5, CXCL6, CXCL9, CXCL10, CXCL11, CXCL12, CXCL13, CXCL16, CX3CL1, macrophage migration inhibitory factor (MIF), Macrophage inflammatory protein (MIP)-1β, soluble CD30 (sCD30), sCD40L, sCD163, chitinase 3-like-1, gp130, IL-1Ra, IL-2Ra, sIL-6Rα, sIL-6Rβ, sTNF-R1, sTNF-R2, TGF-β1, TGF-β2, TGF-β3, APRIL, BAFF, FGF-basic, LIGHT, pentraxin-3, PDGF-BB, RANTES, TRAIL, TSLP, TWEAK, VEGF-A, osteocalcin, osteopontin, matrix metalloproteinase (MMP)-1, MMP-2, and MMP-3.

### 2.4. RNA Sequencing in Peripheral Blood Mononuclear Cells

RNA sequencing in peripheral blood mononuclear cells (PBMCs) was performed in 98 patients whose PBMCs were available for this analysis. In brief, PBMCs were isolated by density gradient centrifugation using Ficoll-Paque Plus (GE Healthcare, Uppsala, Sweden) before initiating ICI therapy and stored frozen until analysis. Total RNA was isolated from PBMCs using the RNeasy mini kit (Qiagen, Hilden, Germany), according to the manufacturer’s instructions. The RNA amount and purity were measured using a NanoDrop 1000 spectrophotometer (Thermo Scientific, Wilmington, DE, USA). The RNA integrity was assessed using the RNA Nano 6000 assay kit with an Agilent Bioanalyzer 2100 system (Agilent Technologies, Santa Clara, CA, USA). Construction of cDNA libraries and RNA sequencing were performed by Takara Bio, Inc. (Kusatsu, Shiga, Japan) using the SMART-Seq^®^ v4 Ultra^®^ Low Input RNA kit (Clontech, Palo Alto, CA, USA) and a NovaSeq sequencing system (Illumina, San Diego, CA, USA) according to the manufacturers’ instructions.

After confirming the quality of the reads with FastQC (https://www.bioinformatics.babraham.ac.uk/projects/fastqc/, accessed on 30 July 2020), transcripts per million (TPM) were calculated using Salmon 1.1.0 (https://combine-lab.github.io/salmon/, accessed on 30 July 2020) [24] with GRCh38.v99 as the reference genome. The Salmon-estimated TPM data were summarized to the gene level using the “tximport” package (v1.22.0) [25] in RStudio for correlation analysis. The obtained read count data for each PBMC sample were analyzed using the Genomon 2 RNA analysis pipeline (https://github.com/Genomon-Project, accessed on 30 July 2020) to check the sequencing quality. The relative abundance of the immune cell types in PBMCs was assessed by CIBERSORT [26].

### 2.5. Statistical Analysis

Progression-free survival (PFS) was defined as the period from the date of the first dose to the date of treatment failure (death or disease progression) or the date of censoring at the final follow-up examination. Overall survival (OS) was defined as the period from the date of the first dose to the date of death from any cause or the date of censoring at the final follow-up examination. The Cox proportional hazards regression model was adopted to evaluate the clinical significance of plasma NET levels and other factors. Data of NET levels were normalized by subtracting the mean and dividing by the standard deviation (SD). First, we evaluated whether they were associated with OS or PFS by univariate analysis. Next, the NET levels and the factors with statistical significance (*p* < 0.05) in the univariate analysis were selected for multivariate analysis to evaluate the influence of each factor on OS or PFS. Kaplan–Meier plots of OS and PFS were also used to estimate the clinical significance of NET levels, and intergroup comparisons were performed using a log-rank test. *p*-values of <0.05 were considered to indicate statistical significance. 

Spearman’s rank correlation coefficient analysis was used to assess the correlations between the NET levels and other soluble immune mediators in plasma or gene expression levels (TPM) or immune-cell compositions in PBMCs. A list of 38 NET-related genes was obtained from the previous study [21] (Table S1). The correlation coefficient (rs) and *p*-value were calculated using cor and cor.test R functions, and the adjusted *p*-value was calculated using the p.adjust R (v. 4.1.3) function by false discovery rate (FDR) using the Benjamini–Hochberg procedure. A correlation coefficient (rs) of >0.3 or <−0.3 with FDR < 0.05 was considered to be statistically correlated. A linear regression model was generated using lm R function after eliminating outliers. The outliers were defined as data points that deviated from the median by more than 1.5 times the interquartile range below the first quartile (Q1) or above the third quartile (Q3). Enrichment analysis with significantly correlated genes (rs > 0.3, FDR < 0.05) was performed using the Metascape database (https://metascape.org/gp/index.html#/main/step1, accessed on 10 February 2024) [27] to assess the pathways potentially relevant to NET formation.

All statistical analyses were conducted using IBM SPSS Statistics (version 19; IBM Corporation, Armonk, NY, USA) and the R programming language (ver. 4.1.3).

## 3. Results

### 3.1. Patient Characteristics

A total of 185 patients with NSCLC who were treated with anti-PD-1 Ab (pembrolizumab or nivolumab) or anti-PD-L1 Ab (atezolizumab) between February 2017 and February 2021 were included (Table 1). The median patient age was 69 (range, 37–96) years. Of the 185 patients, 141 (76.2%) and 44 (23.8%) were male and female, respectively; 66 (35.7%) had a good PS (Eastern Cooperative Oncology Group [ECOG] 0); 152 (82.2%) were current or former smokers; 27 (14.6%) had EGFR, ALK, or ROS1 mutation/rearrangement; 112 (60.6%), 55 (29.7%) and 18 (9.7%) had adenocarcinoma, squamous cell carcinomas, and other types, respectively; 137 (74.1%) and 48 (25.9%) had stage III/IV and recurrent tumors, respectively. Of 164 patients whose tissue samples were available, PD-L1 expression was negative or weak (0%–49% of tumor cells) and strong (≥50% of tumor cells) in 93 (56.7%) and 71 (43.3%) patients, respectively. For treatment, anti-PD-1 Ab (nivolumab or pembrolizumab) and anti-PD-L1 Ab (atezolizumab) were used in 148 (80.0%) and 37 (20.0%) patients, respectively, and chemotherapeutic agents were combined in 65 patients (35.1%). Anti-PD-1 or anti-PD-L1 Ab was administered as the first- and second- or further-line treatment in 92 (49.7%) and 93 (50.3%) patients, respectively. There was no significant difference in pretreatment NET concentrations between males [median (range), 7.52 (0.13–103.45)] and females [median (range), 5.85 (0.30–66.60)] (*p* = 0.111, by *t*-test).

### 3.2. Significant Association of Pretreatment NET Levels with OS in ICI-Treated Patients with NSCLC

**Figure 1:**
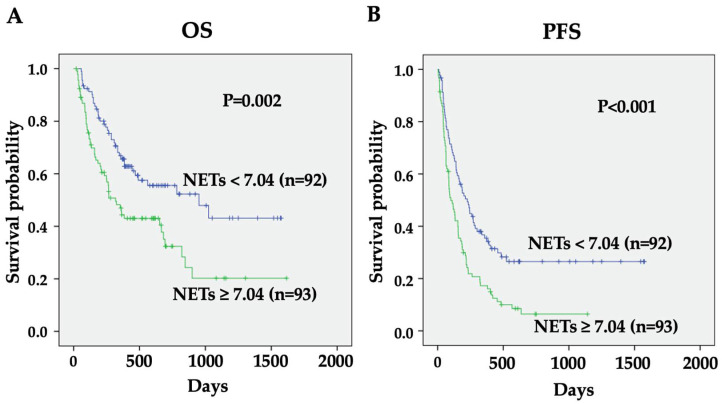
Association between pretreatment NET levels and OS or PFS. Kaplan–Meier plot of OS (**A**) or PFS (**B**) in the high- and low-NET groups. The ICI-treated patients were divided into two groups based on the median value of NETs (7.04 ng/mL). Differences were assessed statistically using the log-rank test. The high-NET group showed significantly worse OS (*p* = 0.002) and PFS (*p* < 0.001) than the low-NET group.

The Cox proportional hazards regression model was used to examine the relationships between OS and pretreatment NET levels or other factors. As shown in Table 2, PS, smoking history, PD-L1 expression, concurrent chemotherapy, treatment line, ALB, LDH, CRP, and pretreatment NET levels were significantly associated with OS (*p* = 0.003, *p* = 0.033, *p* = 0.035, *p* = 0.019, *p* = 0.025, *p* < 0.001, *p* = 0.006, *p* = 0.024, and *p* < 0.001, respectively) by univariate analysis. When the multivariate analysis was performed with the above nine factors, PD-L1 expression (hazard ratio [HR] 0.589, 95% confidence interval [CI] 0.351 to 0.975, *p* = 0.039), ALB (HR 0.397, 95% CI 0.224 to 0.705, *p* = 0.002), and pretreatment NET levels (HR 1.702, 95% CI 1.356 to 2.137, *p* < 0.001) were significantly correlated with OS (Table 2). Kaplan–Meier survival curves were constructed to study the impact of pretreatment NET levels on OS. When the patients were divided into high- and low-NET groups by the median value (7.04 ng/mL) as the cutoff, a significant difference was observed between the high- and low-NET groups (*p* = 0.002) (Figure 1A).

### 3.3. Significant Association of Pretreatment NET Levels with PFS in ICI-Treated Patients with NSCLC

To further evaluate the relationships between PFS and pretreatment NET levels or other factors, similar analyses with the Cox regression model were performed (Table 3). Univariate analysis showed that sex, smoking history, PD-L1 expression, concurrent chemotherapy, treatment line, ALB, and pretreatment NET levels were significantly associated with PFS (*p* = 0.041, *p* = 0.020, *p* = 0.008, *p* = 0.015, *p* = 0.016, *p* = 0.007, and *p* < 0.001, respectively). When the multivariate analysis was performed with the above seven factors, PD-L1 expression (HR 0.539, 95% CI 0.367 to 0.792, *p* = 0.002) and pretreatment NET levels (HR 1.566, 95% CI 1.323 to 1.855, *p* < 0.001) were significantly correlated with PFS. Kaplan–Meier survival curves were constructed to study the impact of pretreatment NET levels on PFS. When the patients were divided into high- and low-NET groups by the median value (7.04 ng/mL) as the cutoff, a significant difference was observed between the high- and low-NET groups (*p* < 0.001) (Figure 1B).

### 3.4. Significant Association of Posttreatment NET Levels with OS and PFS in ICI-Treated Patients with NSCLC

After 6 weeks of ICI treatment, peripheral blood was collected from 164 patients. The Cox proportional hazards regression model was used to examine the relationships between OS and posttreatment NET levels or other factors. As shown in Table S2, PS, smoking history, concurrent chemotherapy, ALB, and posttreatment NET levels were significantly associated with OS by univariate analysis (*p* = 0.019, *p* = 0.007, *p* = 0.030, *p* < 0.001, and *p* < 0.001, respectively). When the multivariate analysis was performed with the above five factors, smoking history (HR 0.540, 95% CI 0.326 to 0.896, *p* = 0.017), ALB (HR 0.457, 95% CI 0.314 to 0.666, *p* < 0.001), and posttreatment NET levels (HR 1.563, 95% CI 1.307 to 1.870, *p* < 0.001) were significantly correlated with OS (Table S2). Kaplan–Meier survival curves were constructed to examine the effect of posttreatment NET levels on OS. When the patients were divided into high- and low-NET groups by the median value (4.82 ng/mL) as the cutoff, a significant difference was observed between the high- and low-NET groups (*p* < 0.001) (Figure S1A).

To further evaluate the relationships between PFS and posttreatment NET levels or other factors, similar analyses were performed using the Cox regression model (Table S3). Univariate analysis showed that sex, smoking history, driver gene mutation, PD-L1 expression, treatment line, and posttreatment NET levels were significantly associated with PFS (*p* = 0.012, *p* = 0.007, *p* = 0.034, *p* = 0.019, *p* = 0.034, and *p* < 0.001, respectively). When the multivariate analysis was performed with the above six factors, sex (HR 1.597, 95% CI 1.057 to 2.411, *p* = 0.026) and posttreatment NET levels (HR 1.442, 95% CI 1.197 to 1.738, *p* < 0.001) were significantly correlated with PFS. Kaplan–Meier survival curves were constructed to examine the impact of posttreatment NET levels on PFS. When the patients were divided into high- and low-NET groups by the median value (4.82 ng/mL) as the cutoff, the low-NET group tended to have better PFS than the high-NET group (*p* = 0.174) (Figure S1B).

These results suggest that NET levels after treatment continued to correlate with patient outcomes.

### 3.5. No Prognostic Significance of Changes in NET Levels after Treatment in ICI-Treated Patients with NSCLC

The Cox proportional hazards regression model was performed to evaluate the prognostic significance of changes in NET levels after treatment. As shown in Table 2 and Table 3, univariate analysis showed that changes in NET levels after treatment were not significantly correlated with OS (*p* = 0.539) or PFS (*p* = 0.621).

### 3.6. Prognostic Significance of Pretreatment NET Levels in Subgroups of ICI-Treated Patients with NSCLC

**Figure 2:**
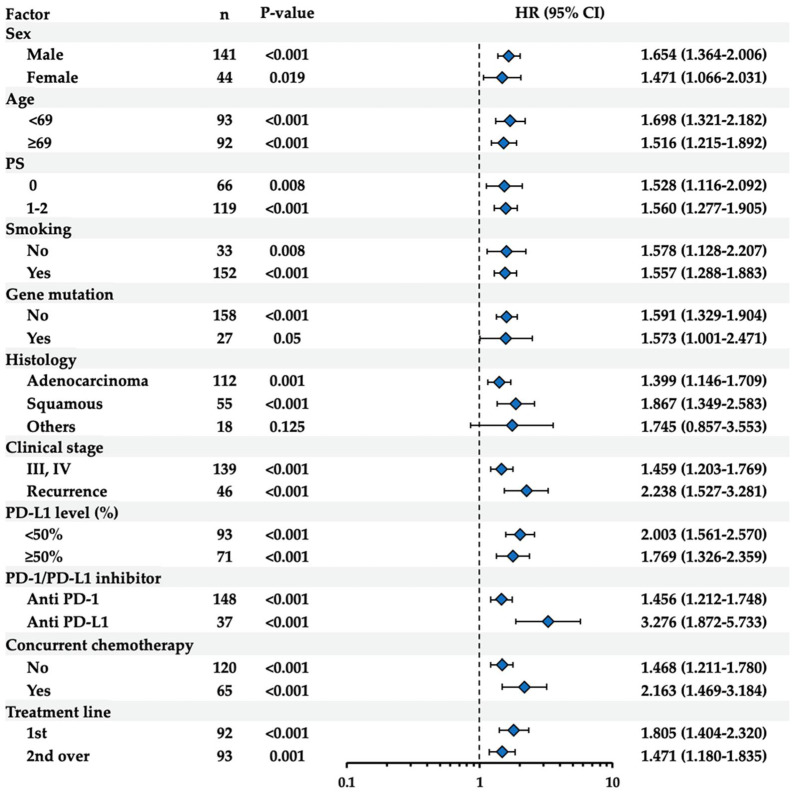
Univariate analysis of OS using Cox regression model with pretreatment NET levels in the subgroups of ICI-treated patients. Results of the univariate analysis in the subgroups stratified by sex, age, PS, smoking history, driver gene mutation, histology, clinical stage, PD-L1 expression levels in tumor tissues, ICI type, concurrent chemotherapy, or treatment line are shown. For age, median values were used as the cutoff. Hazard ratios (HR) and 95% confidence intervals (CI) are shown.

**Figure 3:**
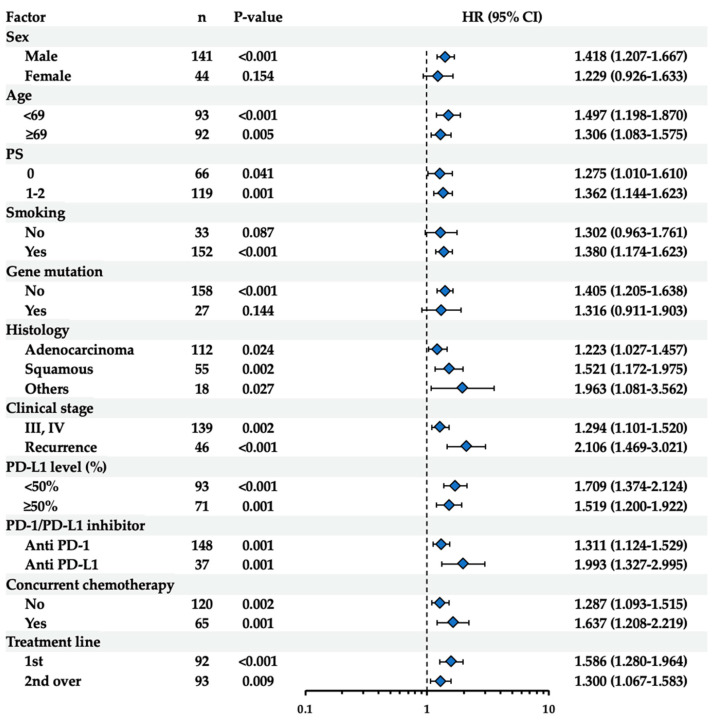
Univariate analysis of PFS using Cox regression model with pretreatment NET levels in the subgroups of ICI-treated patients. Results of the univariate analysis in the subgroups stratified by sex, age, PS, smoking history, driver gene mutation, histology, clinical stage, PD-L1 expression levels in tumor tissues, ICI type, concurrent chemotherapy, or treatment line are shown. For age, median values were used as the cutoff. Hazard ratios (HR) and 95% confidence intervals (CI) are shown.

The clinical significance of pretreatment NET levels was further examined in subgroups stratified by other clinical factors, including sex, age, PS, smoking history, driver gene mutation, histology, clinical stage, PD-L1 expression levels in tumor tissues, ICI type, concurrent chemotherapy, and treatment line. As shown in Figure 2, the pretreatment NET levels were significantly associated with OS in the subgroups examined, except for “others” histology. Similarly, the pretreatment NET levels were also significantly associated with PFS in the subgroups examined, except for females, those with no smoking history, and positive driver gene mutation (Figure 3). Kaplan–Meier survival curves were constructed to study the impact of pretreatment NET levels on OS or PFS in the subgroups based on histopathological types or smoking history. As shown in Figure S2, significant differences were observed between the high- and low-NET groups in the adenocarcinoma (OS: *p* = 0.045; PFS: *p* = 0.008) and squamous cell carcinoma subgroups (OS: *p* = 0.008; PFS: *p* = 0.025), but not in the “others” subgroup (OS: *p* = 0.602; PFS: *p* = 0.117). Additionally, as shown in Figure S3, significant differences were observed between the high- and low-NET groups in the subgroup with smoking history (OS: *p* = 0.021; PFS: *p* < 0.001). In contrast, in the subgroup without a smoking history, the high-NET group was significantly different from the low-NET group in OS (*p* = 0.018) but not in PFS (*p* = 0.353). It may be possible that the small number of patients in the subgroups of females (n = 44), those with no smoking history (n = 33), positive driver gene mutation (n = 27), and “others” histology (n = 18) affected the statistical evaluation in PFS.

### 3.7. Relationships between the Pretreatment NET Levels and Other Soluble Immune Mediators in Plasma and Gene Expression in PBMCs

**Figure 4:**
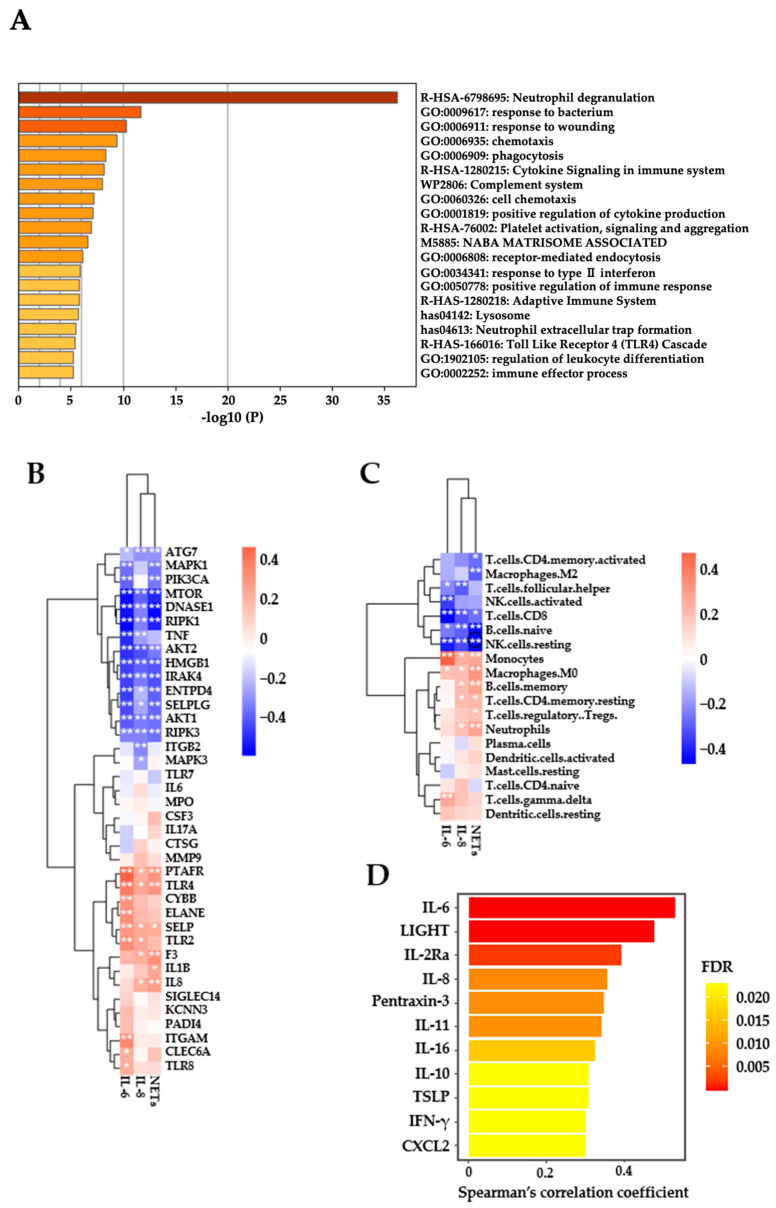

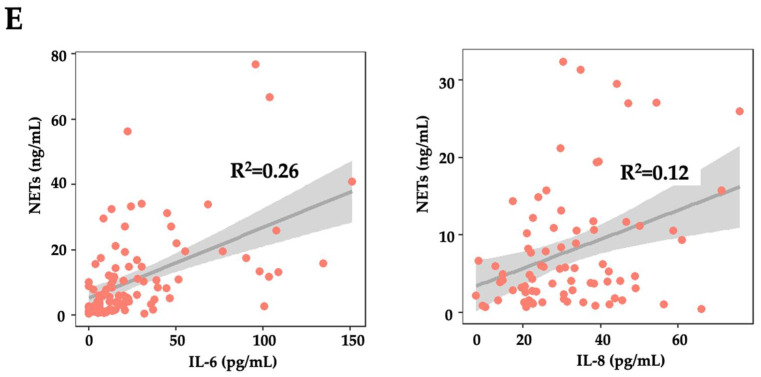
Correlations between the levels of pretreatment NETs and other soluble immune mediators in plasma or gene expression or immune-cell composition in PBMCs. (**A**) Relationships between the pretreatment plasma NET levels and transcriptome in PBMCs from patients whose samples were available for this analysis (n = 98). Enrichment analysis with genes whose expression levels were significantly positively correlated with the NET levels by Spearman’s rank correlation coefficient analysis (rs > 0.3, FDR < 0.05), was performed using the Metascape database. The top 20 enriched pathways are shown. (**B**) Spearman’s rank correlation coefficient analysis between the pretreatment NETs, IL-6, or IL-8 levels in plasma and expression of 38 NET-related genes in PBMCs (n = 98). * *p* < 0.05, ** *p* < 0.01. (**C**) Spearman’s rank correlation coefficient analysis between the pretreatment NETs, IL-6, or IL-8 levels in plasma and frequencies of immune cells assessed by CIBERSORT in PBMCs (n = 98). * *p* < 0.05, ** *p* < 0.01. (**D**) Soluble immune mediators were measured in plasma before ICI treatment in patients whose plasma samples were available for this analysis (n = 98). Their levels were assessed for correlations with the NET levels by Spearman’s rank correlation coefficient analysis. Eleven soluble immune mediators, including IL-6, LIGHT, IL-2Ra, IL-8, pentraxin-3, IL-11, IL-16, IL-10, TSLP, IFNγ, and CXCL2, showed significant correlations (rs > 0.3, FDR < 0.05). (**E**) Correlation between the levels of pretreatment NETs and IL-6 or IL-8 in plasma. The gray line shows the best fit; R2 indicates the adjusted R-square of simple linear regression; the gray shading represents the 95% confidence interval (CI).

To elucidate the potential molecular mechanisms of NET formation, the relationships between the pretreatment NET levels in plasma and gene expression levels in PBMCs, as determined by RNA sequencing, were examined in 98 patients whose PBMCs were available for this analysis. Table S4 shows a list of 189 genes whose expression levels were significantly positively correlated with the NET levels by Spearman’s rank correlation coefficient analysis (rs > 0.3, FDR < 0.05). As shown in Figure 4A, many immune-related pathways were identified by enrichment analysis with these significantly correlated genes. In particular, the neutrophil degranulation pathway, which may play a key role in NET formation, was enriched at the highest level. We further examined the relationship between plasma NET levels and NET-related gene expression or immune-cell composition in PBMCs. As shown in Figure 4B, plasma NET levels showed significantly negative or positive correlations with many NET-related genes [21], suggesting that plasma NETs may be at least partly derived from immune cells in the peripheral blood. Furthermore, plasma NET levels exhibited a negative correlation with the frequencies of activated memory CD4 T cells, macrophage (M2), CD8 T cells, naïve B cells, and resting NK cells, and a positive correlation with those of monocytes, macrophages (M0), memory B cells, resting memory CD4 T cells, regulatory T cells, and neutrophils in PBMCs (Figure 4C). These results suggested that plasma NETs may be associated with immune-suppressive profiles in PBMCs.

The relationships between the pretreatment NET levels and other soluble immune mediators in plasma were also examined in 98 patients whose plasma samples were available for this analysis. The levels of soluble immune mediators in plasma before ICI treatment were assessed for correlations with the NET levels by Spearman’s rank correlation coefficient analysis. As shown in Figure 4D, significant correlations (rs > 0.3, FDR < 0.05) were observed for 11 soluble immune mediators, including IL-6 (rs = 0.531), LIGHT (rs = 0.478), IL-2Ra (rs = 0.394), IL-8 (rs = 0.357), pentraxin-3 (rs = 0.348), IL-11 (rs = 0.342), IL-16 (rs = 0.325), IL-10 (rs = 0.307), TSLP (rs = 0.307), IFNγ (rs = 0.301), and CXCL2 (rs = 0.301). Of note, IL-6 and IL-8, which were reported to be associated with NET formation [20,21,22,23,24,25], showed high linear correlations with the NET levels (Figure 4E). Additionally, plasma IL-6 and IL-8 concentrations showed significantly negative or positive correlations with many NET-related genes (Figure 4B). Of note, plasma IL-8 concentration showed a significantly positive correlation with IL-8 mRNA level in PBMCs (Figure 4B), suggesting that plasma IL-8 may be at least partly derived from immune cells in the peripheral blood. In contrast, plasma IL-6 concentration showed no significant correlation with IL-6 mRNA level in PBMCs (Figure 4B). This result suggested that the production of plasma IL-6 may not be derived from immune cells in the peripheral blood but possibly from other cells or tissues within tumors.

We further examined the correlations between plasma IL-6 or IL-8 concentrations and immune-cell composition in PBMCs. Plasma IL-8 concentration showed a significantly negative correlation with the frequencies of follicular helper T cells, CD8 T cells, naïve B cells, and resting NK cells and a positive correlation with those of monocytes, macrophages (M0), memory B cells, resting memory CD4 T cells, and neutrophils in PBMCs. Similarly, plasma IL-6 concentration showed a significantly negative correlation with the frequencies of follicular helper T cells, CD8 T cells, naïve B cells, and resting and activated NK cells and a positive correlation with those of monocytes, macrophages (M0), and γδ T cells in PBMCs. These results suggest that plasma IL-8 and IL-6 may be associated with immune suppressive profiles in PBMCs. It should be noted that plasma NETs, IL-8, and IL-6 all showed similar correlations with immune-cell compositions in PBMCs.

## 4. Discussion

The presence of NETs in human solid tumors has been reported to be associated with a poor prognosis in various cancers, including NSCLC [11,12,13,14,15,16]. In addition, the roles of NETs in cancer pathogenesis, such as tumor progression, metastasis, and cancer-associated thrombosis, have recently become the focus of attention. Several studies have examined the expression of NET-related genes in tumors from patients with multiple tumor types, including NSCLC [21,28,29], hepatocellular carcinoma [30,31], breast cancer [32], and colon cancer [33], by using large public databases such as The Cancer Genome Atlas (TCGA) and Gene Expression Omnibus (GEO). These studies demonstrated that NET-related gene signatures significantly correlate with the tumor immune microenvironment and poor prognosis in cancer patients. Nevertheless, the clinical roles of NETs in the peripheral blood in patients with cancer who are treated with immunotherapy remain poorly addressed. Wang et al. proposed that a risk score incorporating serum NETs, IL-8, and CRP could serve as a valuable predictive biomarker for ICI therapy in cancer patients [22]. Nevertheless, their study encompassed patients with diverse cancer types, including NSCLC, nasopharyngeal carcinoma, gastrointestinal tumors, and melanoma, each with different clinical characteristics. Additionally, very recently, serum NETs were reported to predict the efficacy of ICI treatment in NSCLC patients, but the study was limited by a small sample size (n = 31) [23]. In this study, we demonstrated that the plasma levels of NETs before and after ICI treatment are associated with OS and PFS in ICI-treated patients with cancer. Notably, the larger sample size (n = 185) in our study might strengthen the validity of the findings. In addition, subgroup analyses demonstrated that the clinical significance of the pretreatment NET levels was not substantially affected by other factors, supporting the reproducibility of our findings. 

Recently, the mechanisms of NET involvement in cancer progression have been extensively examined, especially in mouse models [34,35,36,37,38]. For example, Albrengues et al. [34] reported that NET-associated neutrophil proteases, neutrophil elastase, and MMP-9 were involved in the proteolytic remodeling of laminin and awakened dormant cancer cells. Park et al. [35] demonstrated that metastatic cancer cells induced neutrophils to form NETs, which stimulated tumor invasion and migration, and that DNase I reduced lung metastases by breaking down NETs in mouse breast cancer models. In addition, NETs have been shown to regulate immune cells within the tumor microenvironment and affect the sensitivity of tumor cells to ICIs [17,18,19,20]. For example, Teijieira et al. [17] showed that tumor-secreted CXCR1 and CXCR2 ligands, such as IL-8, induced the extrusion of NETs, which wrapped and coated tumor cells and physically shielded them from CD8+ T cell- and NK cell-mediated cytotoxicity in mice. They also demonstrated that inhibitors of peptidylarginine deaminase 4 (PAD4), which is indispensable for the generation of NETs, sensitized tumors to anti-PD-1 and anti-CTLA-4 dual blockades. Zhang Y. et al. [18] reported that IL-17 recruited neutrophils, triggered the production of NETs, and excluded cytotoxic CD8+ T cells from tumors in a KRAS-induced pancreatic cancer model. At the same time, IL-17 blockades overcame the resistance to PD-1 blockade in a CD8+ T cell-dependent manner. In addition, Zhang H. et al. [19] showed that the DNase I enzyme, which degrades NETs, diminished the resistance to PD-1 blockade by increasing CD8+ T-cell infiltration and cytotoxicity in a mouse colorectal cancer model. Furthermore, Kaltenmeier et al. [20] demonstrated that NETs contained PD-L1, which suppressed T cells through metabolic and functional exhaustion. Consistent with these results, we showed that plasma NETs may be associated with immune suppressive profiles in the peripheral blood in cancer patients. Based on these findings, targeting NETs, together with anti-PD-1 blockade, might be a feasible approach to improve the prognosis in patients with cancer. However, although promising results with NET inhibitors, such as DNase I and PAD4 inhibitors, have been reported in murine tumor models [17,19], no clinical trials in cancer patients have provided conclusive evidence of the efficacy of this strategy. Further preclinical and clinical studies are needed to clarify the therapeutic effects of targeting NETs in cancer patients.

In this study, gene expression analysis in PBMCs showed that the plasma NET levels before ICI treatment were significantly positively or negatively correlated with the expression of NET-related genes in PBMCs, suggesting that plasma NETs may be at least partly derived from immune cells in the peripheral blood. The mechanisms of NET production in patients with cancer have been gradually unraveled. As discussed above, inflammatory cytokines, such as IL-8 and IL-17, were reported to be involved in the production of NETs from neutrophils [17,18,39,40]. In addition, another inflammatory cytokine, IL-6, was also suggested to promote neutrophil activation and NET formation [41]. For example, Thalin et al. [42] showed a positive correlation between plasma IL-6 and IL-8 and NET (CitH3) levels, which were associated with a poor prognosis in patients with cancer. Zhang L. et al. [43] also demonstrated that increased circulating NET levels were positively correlated with the levels of inflammatory cytokines, including IL-1β, IL-6, IL-18, and TNF-α. Similar to these findings, the current study demonstrated that the levels of NETs before ICI treatment were highly correlated with those of IL-6 and IL-8 and that plasma NETs, IL-8, and IL-6 all showed similar correlations with immune-cell compositions in PBMCs. Notably, IL-6 and IL-8 were reported to predict which patients with cancer would not benefit from ICI treatment [44,45,46,47]. This finding might be explained, at least in part, by the ability of IL-6 or IL-8 to trigger the production of NETs. Based on these findings, it is conceivable that the inflammatory environment may activate neutrophils and trigger NET release, thereby reducing the clinical efficacy of ICIs in patients with NSCLC. In the current study, plasma IL-8 concentration showed a significantly positive correlation with IL-8 mRNA expression in PBMCs, suggesting that plasma IL-8 may be at least partly derived from immune cells in the peripheral blood. In contrast, plasma IL-6 concentration showed no significant correlation with IL-6 mRNA expression in PBMCs, indicating that the production of plasma IL-6 may not be derived from immune cells in the peripheral blood but possibly from other cells or tissues within tumors. Further studies are needed to clarify the source of plasma IL-6 and determine the relationship between plasma NETs, IL-6 or IL-8 concentrations and immune-cell compositions in tumors.

In addition to IL-6 and IL-8, significant correlations were observed between pretreatment NET levels and other soluble immune mediators, including LIGHT, IL-2Ra, pentraxin-3, IL-11, IL-16, IL-10, TSLP, IFNγ, and CXCL2. Among them, pentraxin-3 and CXCL2 have also been reported to be potentially associated with NETs. It has been shown that pentraxin-3, an acute phase protein, is stored in specific granules of neutrophils and released in response to inflammatory signals, and that released pentraxin-3 can partially localize to NETs formed by extruded DNA [48,49]. CXCL2 is a ligand of CXCR1 and CXCR2, which can mediate the production of NETs [17]. Therefore, these factors may be important in the production and function of NETs. In contrast, no reports have been published on the relationships of NETs with the other factors, including LIGHT, IL-2Ra, IL-11, IL-16, IL-10, TSLP, and IFNγ. Therefore, further studies are needed to clarify their roles in the production and function of NETs. Interestingly, recent studies have demonstrated sex differences in the phenotype and function of neutrophils. Circulating neutrophils from males were reported to show lower levels of cell-surface maturation markers and lower NET formation at baseline and on stimulation compared to neutrophils from females [50]. However, there was no significant difference in pretreatment NET concentrations between males and females in this study, possibly because plasma NETs are more dependent on other factors than the sex difference.

## 5. Conclusions

This study showed that NET levels before and after ICI treatment were associated with prognosis in ICI-treated patients with NSCLC. Considering the critical roles of NETs in cancer progression, combined treatment with inhibitors of NETs, such as DNase I, could be a promising strategy to improve the efficacy of ICI treatment. Nevertheless, our study had several limitations. First, due to the retrospective study design, selection bias cannot be entirely excluded. Secondly, patients with different clinical characteristics, such as sex, smoking history, driver gene mutations, and type of PD-1/PD-L1 inhibitors, were included, but the number of patients was relatively small to perform detailed subgroup analyses. Third, the effect of plasma NETs on the tumor immune microenvironment remained unclarified since tumor tissues from the enrolled patients were unavailable in this study. Therefore, further large-scale studies are warranted to perform more detailed analyses with blood and tumor samples and to confirm the utility of our findings also in the subgroups of patients with less frequent characteristics, such as females, non-smokers, those with positive driver gene mutations, and those treated with PD-L1 inhibitors. 

## Figures and Tables

**Table 1:** Patient characteristics.

Factor	Patient (n = 185)
Sex, n (%)	
Male	141 (76.2)
Female	44 (23.8)
Age (years), median (range)	69 (37–96)
PS, n (%)	
0	66 (35.7)
1–2	119 (64.3)
Smoking history, n (%)	
No	33 (17.8)
Yes	152 (82.2)
Driver gene mutation (*EGFR*, *ARK*, *ROS1*), n (%)	
No	158 (85.4)
Yes	27 (14.6)
Histology, n (%)	
Adenocarcinoma	112 (60.6)
Squamous	55 (29.7)
Others	18 (9.7)
Clinical stage, n (%)	
III, IV	137 (74.1)
Recurrence	48 (25.9)
PD-L1 level, n (%)	
<50%	93 (56.7)
≥50%	71 (43.3)
ICI type, n (%)	
Anti-PD-1 Ab (Nivolumab, Pembrolizumab)	148 (80.0)
Anti-PD-L1 Ab (Atezolizumab)	37 (20.0)
Concurrent chemotherapy, n (%)	
No	120 (64.9)
Yes	65 (35.1)
Treatment line, n (%)	
1	92 (49.7)
≥2	93 (50.3)
WBC (10^3^/μL), median (range)	6.9 (2.7–36.0)
Plt (10^4^/μL), median (range)	25.2 (5.5–59.6)
ALB (mg/dL), median (range)	3.5 (1.8–4.8)
LDH (U/L), median (range)	207 (121–3069)
CRP (mg/dL), median (range)	0.94 (0.01–20.00)
NETs (ng/mL), median (range)	7.04 (0.13–103.45)
postNETs (ng/mL), median (range)	4.82 (0.37–90.76) [n = 165]
ΔNETs (ng/mL), median (range)	−1.23 (−92.61–49.16) [n = 165]

Categorical variables are shown as the numbers and percentages of patients. Continuous variables are shown as the median and range of values. Abbreviations: PS, performance status; ICI, immune checkpoint inhibitor; Ab, antibody; WBC, white blood cell count; Plt, platelet count; ALB, albumin; LDH, lactate dehydrogenase; CRP, C-reactive protein; NETs, neutrophil extracellular traps before ICI treatment; postNETs, NETs after ICI treatment; ΔNETs, difference in plasma NET concentrations before and after ICI administration.

**Table 2:** Univariate and multivariate Cox proportional hazards analysis of pretreatment NETs and other factors for OS.

Factor	Univariate		Multivariate	
	*p*-Value	HR (95% CI)	*p*-Value	HR (95% CI)
Sex				
female vs. male	0.150	1.382 (0.889–2.149)		
Age (years)	0.503	1.008 (0.985–1.031)		
PS				
1–2 vs. 0	**0.003**	1.995 (1.263–3.151)	0.208	1.434 (0.818–2.513)
Smoking history				
Yes vs. No	**0.033**	0.600 (0.375–0.960)	0.975	0.991 (0.574–1.712)
Driver gene mutation				
Yes vs. No	0.756	0.908 (0.495–1.667)		
Histology				
Squamous vs. Adenocarcinoma	0.772	1.070 (0.678–1.687)		
Others vs. Squamous	0.656	0.836 (0.379–1.841)		
Others vs. Adenocarcinoma	0.661	0.921 (0.636–1.332)		
Clinical stage				
Recurrence vs. III, IV	0.779	0.936 (0.592–1.481)		
PD-L1 level				
≥50% vs. <50%	**0.035**	0.612 (0.388–0.966)	**0.039**	0.589 (0.351–0.975)
ICI type				
Anti-PD-L1 Ab vs. Anti-PD-1 Ab	0.918	1.026 (0.626–1.681)		
Concurrent chemotherapy				
Yes vs. No	**0.019**	0.563 (0.349–0.908)	0.243	0.677 (0.351–1.304)
Treatment line				
≥2 vs. 1	**0.025**	1.602 (1.061–2.418)	0.102	1.544 (0.917–2.599)
WBC (10^3^/μL)	0.088	1.031 (0.996–1.067)		
Plt (10^4^/μL)	0.629	1.005 (0.986–1.024)		
ALB (mg/dL)	**<0.001**	0.387 (0.273–0.550)	**0.002**	0.397 (0.224–0.705)
LDH (U/L)	**0.006**	1.001 (1.000–1.002)	0.151	1.000 (1.000–1.001)
CRP (mg/dL)	**0.024**	1.044 (1.006–1.084)	0.239	0.959 (0.894–1.028)
NETs (ng/mL)	**<0.001**	1.656 (1.388–1.975)	**<0.001**	1.702 (1.356–2.137)
ΔNETs (ng/mL)	0.539	0.995 (0.978–1.005)		

Univariate and multivariate analysis was conducted using the Cox proportional hazard model with pretreatment NETs and other factors for overall survival (OS). Significant *p*-values are shown in bold. Abbreviations: HR, hazard ratio; CI, confidence interval; PS, performance status; ICI, immune checkpoint inhibitor; Ab, antibody; WBC, white blood cell count; Plt, platelet count; ALB, albumin; LDH, lactate dehydrogenase; CRP, C-reactive protein; NETs, neutrophil extracellular traps before ICI treatment; ΔNETs, difference in plasma NET concentrations before and after ICI administration.

**Table 3:** Univariate and multivariate Cox proportional hazards analysis of pretreatment NETs and other factors for PFS.

Factor	Univariate		Multivariate	
	*p*-Value	HR (95% CI)	*p*-Value	HR (95% CI)
Sex				
female vs. male	**0.041**	1.457 (1.015–2.092)	0.278	1.345 (0.787–2.301)
Age (years)	0.798	1.002 (0.984–1.021)		
PS				
1–2 vs. 0	0.403	1.157 (0.822–1.628)		
Smoking history				
Yes vs. No	**0.020**	0.626 (0.422–0.929)	0.606	1.174 (0.638–2.159)
Driver gene mutation				
Yes vs. No	0.090	1.453 (0.944–2.236)		
Histology				
Squamous vs. Adenocarcinoma	0.485	0.878 (0.608–1.266)		
Others vs. Squamous	0.305	0.705 (0.362–1.375)		
Others vs. Adenocarcinoma	0.113	0.776 (0.568–1.062)		
Clinical stage				
Recurrence vs. III, IV	0.936	1.015 (0.699–1.475)		
PD-L1 level				
≥50% vs. <50%	**0.008**	0.620 (0.435–0.884)	**0.002**	0.539 (0.367–0.792)
ICI type				
Anti-PD-L1 Ab vs. Anti-PD-1 Ab	0.431	1.167 (0.795–1.714)		
Concurrent chemotherapy				
Yes vs. No	**0.015**	0.651 (0.461–0.919)	0.050	0.637 (0.405–1.001)
Treatment line				
≥2 vs. 1	**0.016**	1.492 (1.077–2.065)	0.097	1.443 (0.935–2.227)
WBC (10^3^/μL)	0.457	1.013 (0.979–1.047)		
Plt (10^4^/μL)	0.177	0.989 (0.974–1.005)		
ALB (mg/dL)	**0.007**	0.678 (0.511–0.899)	0.096	0.742 (0.522–1.054)
LDH (U/L)	0.253	1.000 (1.000–1.001)		
CRP (mg/dL)	0.433	1.013 (0.981–1.046)		
NETs (ng/mL)	**<0.001**	1.381 (1.194–1.597)	**<0.001**	1.566 (1.323–1.855)
ΔNETs (ng/mL)	0.621	0.997 (0.985–1.009)		

Univariate and multivariate analysis was conducted using the Cox proportional hazard model with pretreatment NETs and other factors for progression-free survival (PFS). Significant *p*-values are shown in bold. Abbreviations: HR, hazard ratio; CI, confidence interval; PS, performance status; ICI, immune checkpoint inhibitor; Ab, antibody; WBC, white blood cell count; Plt, platelet count; ALB, albumin; LDH, lactate dehydrogenase; CRP, C-reactive protein; NETs, neutrophil extracellular traps before ICI treatment; ΔNETs, difference in plasma NET concentrations before and after ICI administration.

## Data Availability

Data are available upon reasonable request to the corresponding author; the request must include a description of the research proposal.

## Supplementary Materials

The following supporting information can be downloaded at https://www.mdpi.com/article/10.3390/biomedicines12081831/s1, Table S1: List of 38 NET-related genes. Table S2: Univariate and multivariate Cox proportional hazards analysis of posttreatment NETs and other factors for OS. Table S3: Univariate and multivariate Cox proportional hazards analyses of posttreatment NETs and other factors for PFS. Table S4: List of genes positively correlated with the pretreatment NET levels in plasma. Figure S1: Association between posttreatment NET levels and OS or PFS. Figure S2: Association between pretreatment NET levels and OS or PFS in the subgroups based on histopathological types. Figure S3: Association between pretreatment NET levels and OS or PFS in the subgroups based on smoking history.
