# Supplementary material for: Prognostic Significance of Plasma Neutrophil Extracellular Trap Levels in Patients with Non-Small Cell Lung Cancer Treated with Immune Checkpoint Inhibitors
Source: Biomedicines. 2024 Aug 12;12(8):1831. doi: 10.3390/biomedicines12081831 (PMC11351864; doi:10.3390/biomedicines12081831)
Supplement: Supplementary file 1 [file biomedicines-12-01831-s001.zip › Supplementary Figures.pdf]

Figure S1

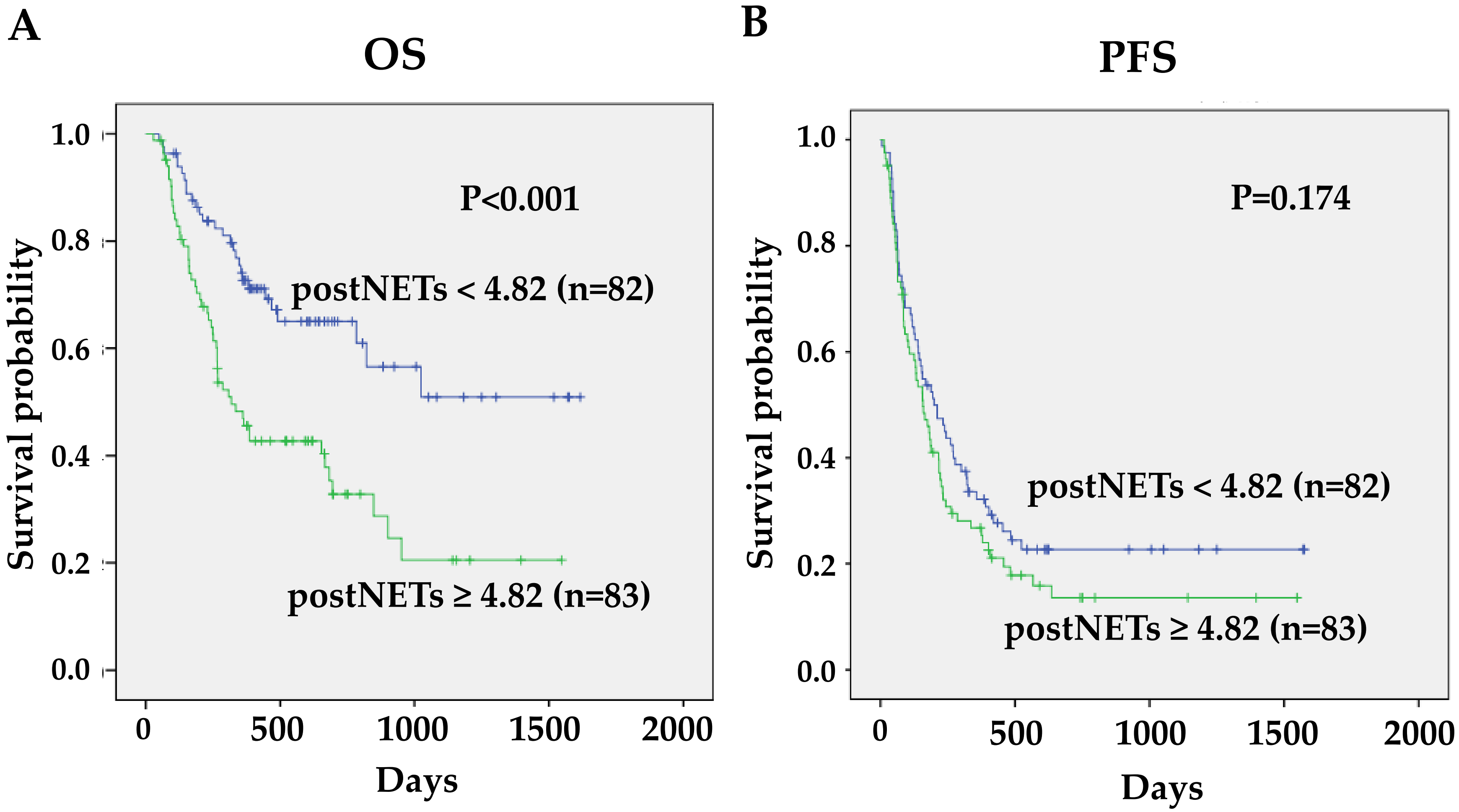

Figure S1. Association between posttreatment NET levels and OS or PFS. Kaplan–Meier plot of OS (A) or PFS (B) in the high- and low-NET groups. The ICI-treated patients were divided into two groups based on the median value of postNETs (4.82 ng/mL). Differences were assessed statistically using the log-rank test.

Figure S2

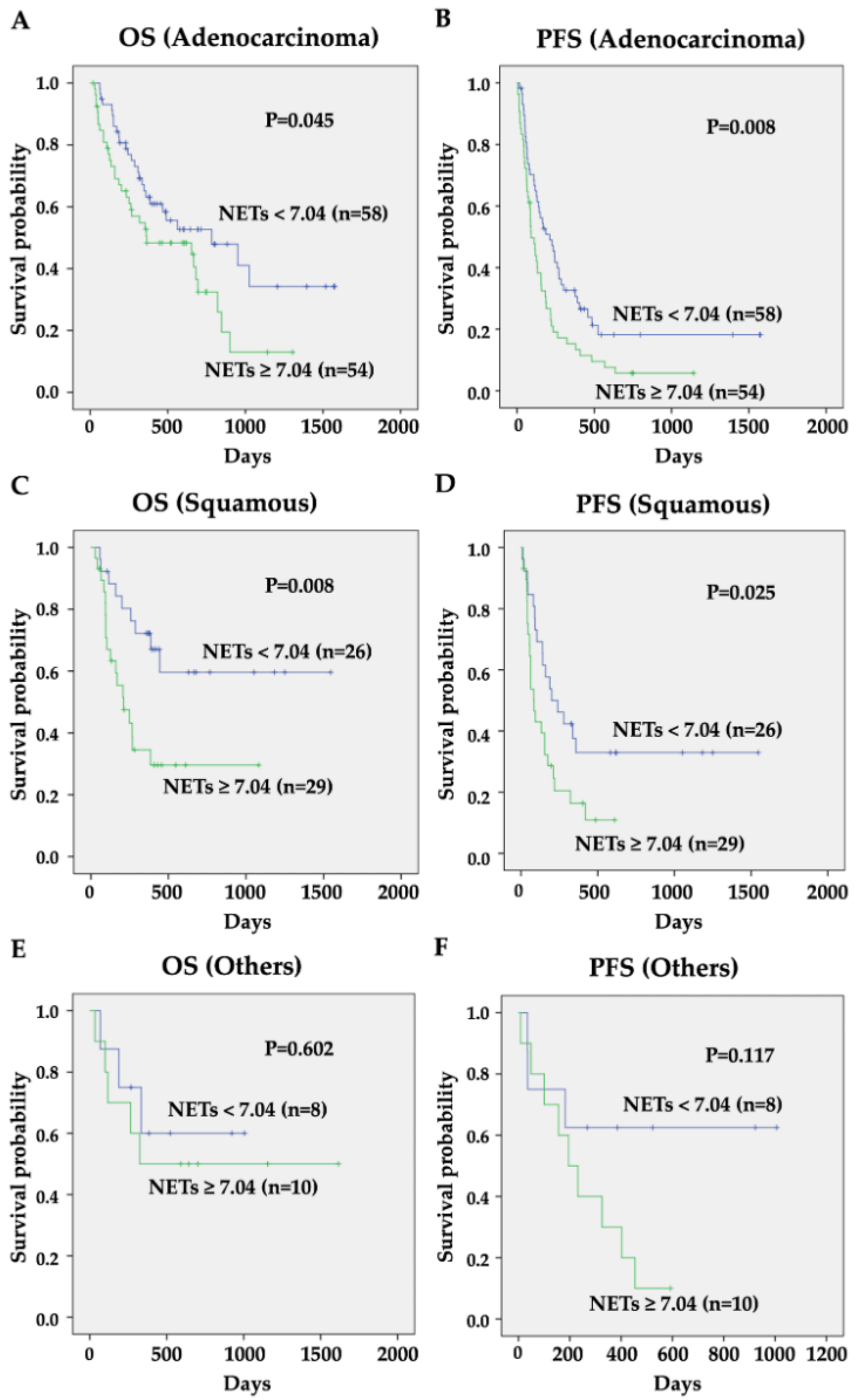

Figure S2. Association between pretreatment NET levels and OS or PFS in the subgroups based on histopathological types. Kaplan–Meier plot of OS or PFS in the high- and low-NET groups in the subgroup of adenocarcinoma (A, B), squamous cell carcinoma (C, D), and others (E,F). The ICI-treated patients were divided into two groups based on the median value of NETs (7.04 ng/mL). Differences were assessed statistically using the log-rank test.

Figure S3

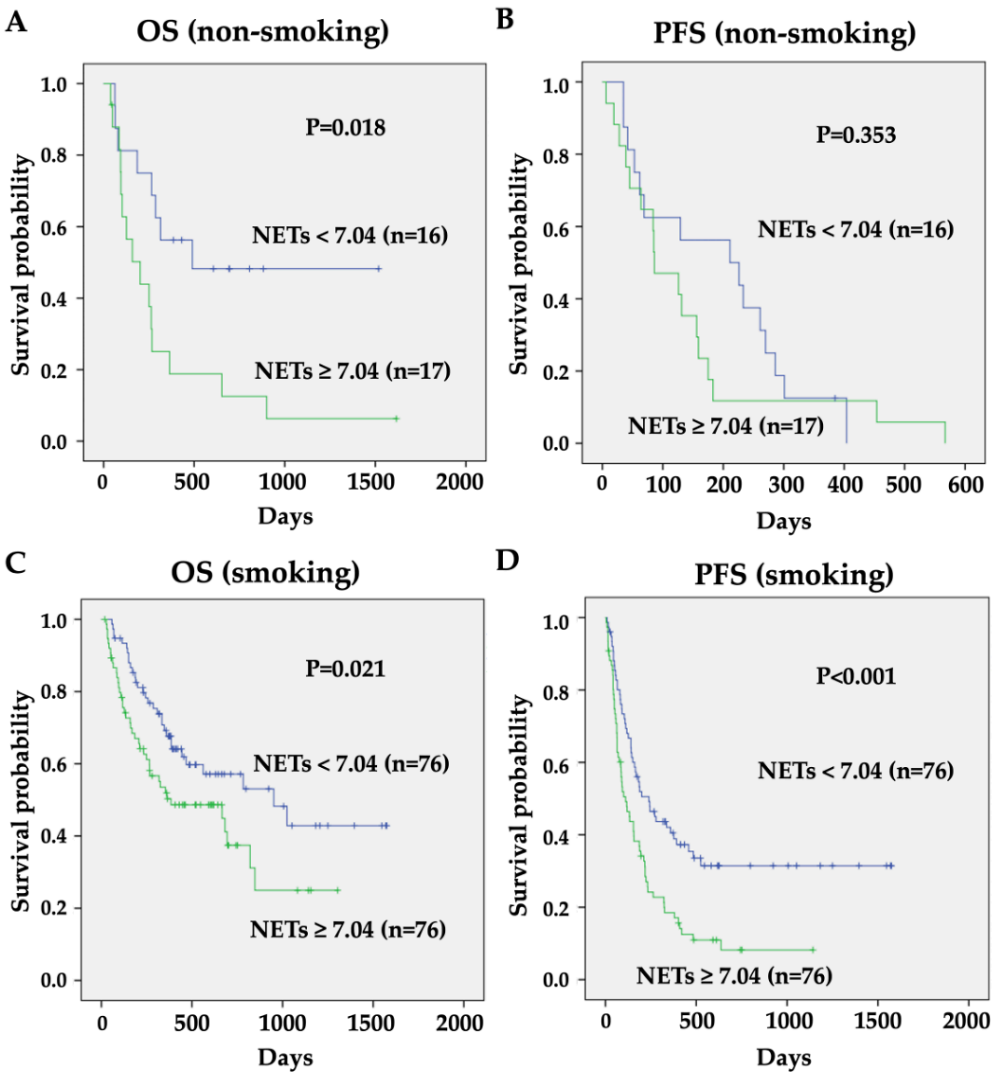

Figure S3. Association between pretreatment NET levels and OS or PFS in the subgroups based on smoking history. Kaplan–Meier plot of OS or PFS in the high- and low-NET groups in the subgroup without (A, B) and with (C, D) smoking history. The ICI-treated patients were divided into two groups based on the median value of NETs (7.04 ng/mL). Differences were assessed statistically using the log-rank test.
