# Supplementary material for: Prognostic Significance of Plasma Neutrophil Extracellular Trap Levels in Patients with Non-Small Cell Lung Cancer Treated with Immune Checkpoint Inhibitors
Source: Biomedicines. 2024 Aug 12;12(8):1831. doi: 10.3390/biomedicines12081831 (PMC11351864; doi:10.3390/biomedicines12081831)
Supplement: Supplementary file 1 [file biomedicines-12-01831-s001.zip › Supplementary Tables.pdf]

Table S1. List of 38 NET-related genes.

Gene name	Ensembl	Description
AKT1	ENSG00000142208	AKT serine/threonine kinase 1
AKT2	ENSG00000105221	AKT serine/threonine kinase 2
ATG7	ENSG00000197548	Autophagy related 7
CLEC6A	ENSG00000205846	Dectin-2
CSF3	ENSG00000108342	Granulocyte colony stimulating factor
CTSG	ENSG00000100448	Cathepsin G
CYBB	ENSG00000165168	NADPH oxidase
DNASE1	ENSG00000213938	Deoxyribonuclease I
ELANE	ENSG00000197561	Neutrophil elastase
ENTPD4	ENSG00000197217	Ectonucleoside Triphosphate Diphosphohydrolase 4
F3	ENSG00000117525	Coagulation Factor III, tissue factor
HMGB1	ENSG00000189403	High mobility group box 1
IL17A	ENSG00000112115	Interleukin 17
IL1B	ENSG00000125538	Interleukin 1 beta
IL6	ENSG00000136244	Interleukin 6
IL8	ENSG00000169429	Interleukin 8
IRAK4	ENSG00000198001	Interleukin 1 receptor associated kinase 4
ITGAM	ENSG00000169896	Complement component 3 receptor 3 subunit
ITGB2	ENSG00000160255	Complement component 3 receptor 3 and 4 subunit
KCNN3	ENSG00000143603	Potassium channel, calcium activated
MAPK1	ENSG00000100030	Mitogen-activated protein kinase 1
MAPK3	ENSG00000102882	Mitogen-activated protein kinase 3
MMP9	ENSG00000100985	Matrix metallopeptidase 9
MPO	ENSG00000005381	Myeloperoxidase
MTOR	ENSG00000198793	Mechanistic target of rapamycin kinase
PADI4	ENSG00000159339	Peptidyl arginine deiminase 4
PTAFR	ENSG00000169403	Platelet activation factor receptor
PIK3CA	ENSG00000121879	Phosphatidylinositol-4,5-bisphosphate 3-kinase
RIPK1	ENSG00000137275	Receptor interacting serine/threonine kinase 1
RIPK3	ENSG00000129465	Receptor interacting serine/threonine kinase 3
SELP	ENSG00000174175	P-selectin
SELPLG	ENSG00000110876	P-selectin receptor
SIGLEC14	ENSG00000254415	Sialic acid binding Ig like lectin 4
TLR2	ENSG00000137462	Toll like receptor 2
TLR4	ENSG00000136869	Toll like receptor 4
TLR7	ENSG00000196664	Toll like receptor 7
TLR8	ENSG00000101916	Toll like receptor 8
TNF	ENSG00000232810	Tumor necrosis factor-alpha

Table S2. Univariate and multivariate Cox proportional hazards analysis of posttreatment NETs and other factors for OS

**Factor**	**Univariate**		**Multivariate**	
	**p-value**	**HR (95% CI)**	**p-value**	**HR (95% CI)**
Sex				
female vs male	0.068	1.547 (0.969–2.471)		
Age (years)	0.392	1.011 (0.986–1.037)		
PS				
1-2 vs 0	**0.019**	1.784 (1.099–2.897)	0.199	1.391 (0.841–2.300)
Smoking history				
Yes vs No	**0.007**	0.511 (0.312–0.835)	**0.017**	0.540 (0.326–0.896)
Driver gene mutation				
Yes vs No	0.954	0.981 (0.518–1.858)		
Histology				
Squamous vs Adenocarcinoma	0.871	1.042 (0.631–1.720)		
Others vs Squamous	0.714	0.853 (0.364–1.998)		
Others vs Adenocarcinoma	0.678	0.920 (0.619–1.366)		
Clinical stage				
Recurrence vs III, IV	0.952	0.985 (0.602–1.612)		
PD-L1 level				
≥50% vs <50%	0.100	0.657 (0.399–1.083)		
ICI type				
Anti-PD-L1 Ab vs Anti-PD-1 Ab	0.970	1.010 (0.591–1.727)		
Concurrent chemotherapy				
Yes vs No	**0.030**	0.564 (0.336–0.947)	0.199	0.706 (0.415–1.201)
Treatment line				
≥2 vs 1	0.069	1.513 (0.968–2.366)		
WBC (10^3/μL)	0.223	1.025 (0.985–1.066)		
Plt (10^4/μL)	0.528	1.006 (0.986–1.027)		
ALB (mg/dL)	**<0.001**	0.404 (0.276–0.593)	**<0.001**	0.457 (0.314–0.666)
LDH (U/L)	0.592	1.000 (0.999–1.002)		
CRP (mg/dL)	0.167	1.031 (0.987–1.076)		
postNETs (ng/mL)	**<0.001**	1.537 (1.294–1.826)	**<0.001**	1.563 (1.307–1.870)

Univariate and multivariate analysis was conducted using the Cox proportional hazard model with posttreatment NETs and other factors for overall survival (OS). Significant p-values are shown in bold.

Abbreviations: HR, hazard ratio; CI, confidence interval; PS, performance status; ICI, immune checkpoint inhibitor; Ab, antibody; WBC, white blood cell count; Plt, platelet count; ALB, albumin; LDH, lactate dehydrogenase; CRP, C-reactive protein; postNETs, neutrophil extracellular traps after ICI treatment.

Table S3. Univariate and multivariate Cox proportional hazards analysis of posttreatment NETs and other factors for PFS

Factor	Univariate		Multivariate	
	**p-value**	**HR (95% CI)**	**p-value**	**HR (95% CI)**
Sex				
female vs male	**0.012**	1.622 (1.111–2.368)	**0.026**	1.597 (1.057–2.411)
Age (years)	0.624	1.005 (0.985–1.025)		
PS				
1-2 vs 0	0.895	1.025 (0.715–1.469)		
Smoking history				
Yes vs No	**0.007**	0.568 (0.375–0.858)	0.606	0.843 (0.441–1.612)
Driver gene mutation				
Yes vs No	**0.034**	1.617 (1.036–2.523)	0.137	1.471 (0.885–2.445)
Histology				
Squamous vs Adenocarcinoma	0.483	0.868 (0.585–1.289)		
Others vs Squamous	0.344	0.712 (0.352–1.439)		
Others vs Adenocarcinoma	0.127	0.774 (0.557–1.075)		
Clinical stage				
Recurrence vs III, IV	0.849	1.039 (0.699–1.546)		
PD-L1 level				
≥50% vs <50%	**0.019**	0.632 (0.432–0.927)	0.067	0.697 (0.474–1.026)
ICI type				
Anti-PD-L1 Ab vs Anti-PD-1 Ab	0.409	1.188 (0.789–1.790)		
Concurrent chemotherapy				
Yes vs No	0.051	0.696 (0.484–1.001)		
Treatment line				
≥2 vs 1	**0.034**	1.458 (1.030–2.065)	0.168	1.312 (0.892–1.928)
WBC (10^3/μL)	0.681	1.008 (0.971–1.046)		
Plt (10^4/μL)	0.208	0.990 (0.973–1.006)		
ALB (mg/dL)	0.093	0.769 (0.566–1.045)		
LDH (U/L)	0.798	1.000 (0.999–1.001)		
CRP (mg/dL)	0.960	1.001 (0.966–1.037)		
postNETs (ng/mL)	**<0.001**	1.443 (1.212–1.719)	**<0.001**	1.442 (1.197–1.738)

Univariate and multivariate analysis was conducted using the Cox proportional hazard model with posttreatment NETs and other factors for progression-free survival (PFS). Significant p-values are shown in bold.

Abbreviations: HR, hazard ratio; CI, confidence interval; PS, performance status; ICI, immune checkpoint inhibitor; Ab, antibody; WBC, white blood cell count; Plt, platelet count; ALB, albumin; LDH, lactate dehydrogenase; CRP, C-reactive protein; postNETs, neutrophil extracellular traps after ICI treatment.

Table S4. List of genes positively correlated with the pretreatment NET levels in plasma

Gene name	Correlation coefficient (*rs*)	p-value	FDR
NAMPT	0.542	1.E-08	3.E-06
GNG10	0.515	9.E-08	8.E-06
MXD1	0.512	1.E-07	9.E-06
SRGN	0.508	1.E-07	1.E-05
S100A12	0.503	2.E-07	1.E-05
TMEM158	0.488	5.E-07	2.E-05
ADM	0.488	5.E-07	2.E-05
DNAJC25-GNG10	0.486	6.E-07	2.E-05
YBX3	0.480	8.E-07	3.E-05
AQP9	0.479	8.E-07	3.E-05
HLX	0.475	1.E-06	3.E-05
TREM1	0.471	1.E-06	3.E-05
CD63	0.468	2.E-06	4.E-05
S100A9	0.465	2.E-06	4.E-05
NAMPTP1	0.464	2.E-06	4.E-05
MAFB	0.463	2.E-06	5.E-05
CXCL16	0.460	2.E-06	5.E-05
CCR1	0.460	3.E-06	5.E-05
LRG1	0.458	3.E-06	5.E-05
SOD2	0.457	3.E-06	5.E-05
ASGR2	0.455	3.E-06	6.E-05
FPR2	0.454	3.E-06	6.E-05
ETF1	0.454	4.E-06	6.E-05
THBD	0.445	6.E-06	8.E-05
S100A8	0.440	7.E-06	1.E-04
CLU	0.438	8.E-06	1.E-04
BCL2A1	0.436	9.E-06	1.E-04
TESC	0.435	9.E-06	1.E-04
BCL3	0.433	1.E-05	1.E-04
FPR1	0.432	1.E-05	1.E-04
UBE2D3	0.431	1.E-05	1.E-04
SLC2A3	0.431	1.E-05	1.E-04
TRIB1	0.431	1.E-05	1.E-04
CEACAM4	0.429	1.E-05	1.E-04
IMPA2	0.426	2.E-05	2.E-04
GAS6	0.424	2.E-05	2.E-04
H3P6	0.420	2.E-05	2.E-04
FAM89A	0.418	2.E-05	2.E-04
UBE2J1	0.418	2.E-05	2.E-04
UBTD1	0.416	2.E-05	2.E-04
F13A1	0.416	2.E-05	2.E-04
ADIPOR1	0.414	3.E-05	2.E-04
RNASE2	0.412	3.E-05	2.E-04
MYL9	0.409	3.E-05	3.E-04
CTSA	0.407	4.E-05	3.E-04
NFIL3	0.406	4.E-05	3.E-04
GNA15	0.403	4.E-05	3.E-04
PDLIM7	0.401	5.E-05	3.E-04
TLE3	0.394	7.E-05	4.E-04
FLVCR2	0.391	8.E-05	5.E-04
SRXN1	0.391	8.E-05	5.E-04
B3GNT5	0.390	8.E-05	5.E-04
CD14	0.389	8.E-05	5.E-04
FCGR1A	0.389	9.E-05	5.E-04
LAPTM4A	0.388	9.E-05	5.E-04
SLC12A9	0.387	1.E-04	6.E-04
PROK2	0.386	1.E-04	6.E-04

OTUD1	0.385	1.E-04	6.E-04
TIMP1	0.384	1.E-04	6.E-04
MCEMP1	0.384	1.E-04	6.E-04
CPNE2	0.383	1.E-04	6.E-04
ZYX	0.381	1.E-04	7.E-04
FLOT1	0.380	1.E-04	7.E-04
DSE	0.380	1.E-04	7.E-04
ACPP	0.380	1.E-04	7.E-04
CKAP4	0.379	1.E-04	7.E-04
EAF1	0.379	1.E-04	7.E-04
PGAM1	0.378	1.E-04	7.E-04
PLAUR	0.378	1.E-04	8.E-04
SHISA5	0.377	1.E-04	8.E-04
CMTM6	0.377	1.E-04	8.E-04
PLK3	0.377	1.E-04	8.E-04
MIR22HG	0.376	2.E-04	8.E-04
SMCO4	0.372	2.E-04	9.E-04
MCL1	0.371	2.E-04	9.E-04
EMC3	0.370	2.E-04	1.E-03
GAPDH	0.369	2.E-04	1.E-03
HCK	0.369	2.E-04	1.E-03
SLC22A4	0.368	2.E-04	1.E-03
PLIN3	0.368	2.E-04	1.E-03
SERPINB1	0.367	2.E-04	1.E-03
ITGA2B	0.366	2.E-04	1.E-03
PLBD1-AS1	0.366	2.E-04	1.E-03
GLT1D1	0.365	2.E-04	1.E-03
BASP1	0.364	3.E-04	1.E-03
HBEGF	0.363	3.E-04	1.E-03
DUSP3	0.362	3.E-04	1.E-03
LDLRAD3	0.362	3.E-04	1.E-03
GCA	0.362	3.E-04	1.E-03
FCGR2A	0.361	3.E-04	1.E-03
FAM53C	0.360	3.E-04	1.E-03
DYSF	0.358	3.E-04	1.E-03
SGK1	0.358	3.E-04	1.E-03
FCN1	0.357	3.E-04	1.E-03
LRRC59	0.356	4.E-04	2.E-03
TALDO1	0.355	4.E-04	2.E-03
PLBD1	0.355	4.E-04	2.E-03
BCAT1	0.354	4.E-04	2.E-03
NCF4	0.354	4.E-04	2.E-03
GPR27	0.353	4.E-04	2.E-03
ACTN1	0.353	4.E-04	2.E-03
SLC25A37	0.352	4.E-04	2.E-03
RAB13	0.350	4.E-04	2.E-03
CARD19	0.350	4.E-04	2.E-03
SPA17	0.349	5.E-04	2.E-03
S100A11	0.349	5.E-04	2.E-03
CPVL	0.348	5.E-04	2.E-03
HMGB3	0.348	5.E-04	2.E-03
VIM	0.346	5.E-04	2.E-03
PIM3	0.345	5.E-04	2.E-03
OSCAR	0.345	5.E-04	2.E-03
CREG1	0.345	5.E-04	2.E-03
SLC38A10	0.344	6.E-04	2.E-03
CREB5	0.344	6.E-04	2.E-03
RER1	0.344	6.E-04	2.E-03
PLB1	0.343	6.E-04	2.E-03
GP1BB	0.343	6.E-04	2.E-03

GADD45G	0.343	6.E-04	2.E-03
CLEC4D	0.342	6.E-04	2.E-03
NIBAN2	0.341	6.E-04	2.E-03
PGD	0.341	6.E-04	2.E-03
PYGL	0.341	6.E-04	2.E-03
ASAH1	0.341	6.E-04	2.E-03
GPCPD1	0.338	7.E-04	3.E-03
STAB1	0.334	8.E-04	3.E-03
TMEM167A	0.333	8.E-04	3.E-03
BNIP3L	0.333	8.E-04	3.E-03
PNP	0.331	9.E-04	3.E-03
NECTIN2	0.330	1.E-03	3.E-03
VAPA	0.330	1.E-03	3.E-03
PTAFR	0.329	1.E-03	3.E-03
ANPEP	0.329	1.E-03	3.E-03
WSB2	0.329	1.E-03	3.E-03
CD93	0.327	1.E-03	4.E-03
MSRB2	0.327	1.E-03	4.E-03
TLR4	0.327	1.E-03	4.E-03
ACSL1	0.326	1.E-03	4.E-03
SH3BGRL3	0.326	1.E-03	4.E-03
CHMP2A	0.325	1.E-03	4.E-03
C1orf198	0.325	1.E-03	4.E-03
SORT1	0.325	1.E-03	4.E-03
H2AC19	0.324	1.E-03	4.E-03
CSRNP1	0.323	1.E-03	4.E-03
SIGLEC5	0.322	1.E-03	4.E-03
MMP25	0.322	1.E-03	4.E-03
GNAQ	0.321	1.E-03	4.E-03
CEBPB	0.321	1.E-03	4.E-03
S100A6	0.321	1.E-03	4.E-03
RIOK3	0.320	1.E-03	5.E-03
METTL9	0.320	1.E-03	5.E-03
LAMTOR1	0.319	1.E-03	5.E-03
H1-0	0.319	1.E-03	5.E-03
CTSD	0.318	1.E-03	5.E-03
CHP1	0.317	2.E-03	5.E-03
RAB31	0.317	2.E-03	5.E-03
SOCS3	0.316	2.E-03	5.E-03
IFI30	0.316	2.E-03	5.E-03
EHD4	0.316	2.E-03	5.E-03
NAIP	0.315	2.E-03	5.E-03
C3AR1	0.315	2.E-03	5.E-03
VNN2	0.315	2.E-03	5.E-03
ATP6V0D1	0.315	2.E-03	5.E-03
PTTG1IP	0.315	2.E-03	5.E-03
RAB20	0.314	2.E-03	5.E-03
SERPINB2	0.314	2.E-03	5.E-03
LTA4H	0.313	2.E-03	6.E-03
NAIPP1	0.313	2.E-03	6.E-03
LRPAP1	0.312	2.E-03	6.E-03
SMIM3	0.312	2.E-03	6.E-03
LILRA5	0.312	2.E-03	6.E-03
STK17B	0.311	2.E-03	6.E-03
FTH1	0.310	2.E-03	6.E-03
EGR3	0.309	2.E-03	6.E-03
S100P	0.309	2.E-03	6.E-03
GSTO1	0.308	2.E-03	6.E-03
SHKBP1	0.306	2.E-03	7.E-03
CD300C	0.306	2.E-03	7.E-03

SLC11A1	0.306	2.E-03	7.E-03
BACH1	0.306	2.E-03	7.E-03
LINC00482	0.306	2.E-03	7.E-03
DGAT2	0.305	2.E-03	7.E-03
CXCL8	0.305	2.E-03	7.E-03
CDA	0.304	2.E-03	7.E-03
ADAM17	0.303	3.E-03	7.E-03
MS4A4A	0.302	3.E-03	8.E-03
CSTA	0.301	3.E-03	8.E-03
MSRB1	0.301	3.E-03	8.E-03
RNF141	0.301	3.E-03	8.E-03
DUSP6	0.300	3.E-03	8.E-03

Correlations between the pretreatment NET levels in plasma and gene expression levels (TPM) by RNA sequencing in PBMCs were analyzed by using Spearman's rank correlation coefficient analysis. The correlation coefficient (*rs*), p-value, and false discovery rate (FDR) are shown.
Abbreviations: NET, neutrophil extracellular trap.
